# Two Cases of Small Cell Carcinoma of the Gallbladder

**DOI:** 10.1155/2010/453624

**Published:** 2010-10-10

**Authors:** Peter Nau, James Liu, Mary Dillhoff, Meghan Forster, Jeffrey Hazey, Scott Melvin

**Affiliations:** Department of General Surgery, Center for Minimally Invasive Surgery, The Ohio State University Medical Center, Columbus, OH 43210, USA

## Abstract

Small cell carcinoma of the gallbladder is a rare disease process with approximately 40 cases reported in the literature. It is most often found in elderly female population and is associated with cholelithiasis and cigarette smoking. A multidisciplinary approach to treatment with wide surgical resection and adjuvant chemotherapy is the current standard of care. Notwithstanding prompt medical intervention, it is a disease with a poor prognosis. The pathology is characterized by early metastases and extensive local invasion. Herein, we report two cases of small cell carcinoma addressed at our institution. In both cases, a radical resection was performed with subsequent referral to oncology for additional therapy.

## 1. Introduction

Approximately 7000 new cases of gallbladder carcinoma are diagnosed every year [1]. It is a disease process characterized by its insidious onset and advanced stage at presentation, often rendering resection impossible and resulting in an overall median survival of six months [2]. First described in 1981, gallbladder neuroendocrine carcinomas make up a small fraction of this already uncommon tumor [3]. These tumors may be secretory with symptoms arising due to the production of biologically active peptides [4]. Conversely, they can be nonsecretory with symptoms presenting due to mass effect and disease progression. The overall five-year survival rate for gallbladder cancer is approximately five percent [5]. The survival rate for neuroendocrine carcinomas is even less due to its high malignant potential and late stage at presentation [6, 7]. Herein, we report two cases of neuroendocrine carcinoma diagnosed and resected in otherwise healthy individuals.

## 2. Case 1

**Figure 1:**
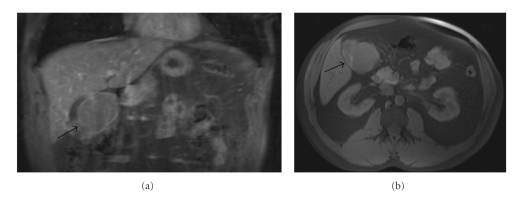
Neuroendocrine tumor of the gallbladder in an otherwise asymptomatic 47-year-old male. (a) Coronal T1-weighted image shows a 7.5 × 5.4 cm irregular mass arising from the gallbladder bed. (b) Coronal T1-weight image again illustrating the large heterogeneous mass in the gallbladder fossa.

A-47-year-old male presented to an outside hospital with a 48-hour history of epigastric and right-upper quadrant abdominal pain. A review of systems was grossly negative at presentation. An RUQ ultrasound and MRI performed revealed a heterogeneous mass arising from the gallbladder measuring 7.5 × 5.4 cm and extending into segment 4B of the liver (Figure 1). Liver function tests, CEA, and CA19-9 were remarkable only for a mildly elevated GGT of 84.

**Figure 2:**
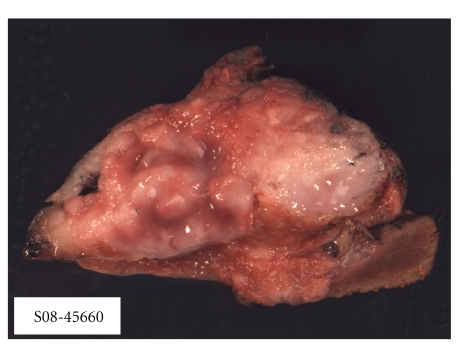
Grossly, the intramural tumor mass occupies over 95% of the gallbladder wall and protrudes into the lumen. The tumor mass has a solid tan-red colored fleshy cut surface.

**Figure 3:**
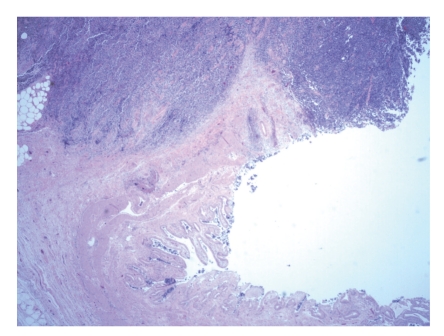
The tumor diffusely infiltrates into the gallbladder wall and involves the mucosa. (HE) ×20.

**Figure 4:**
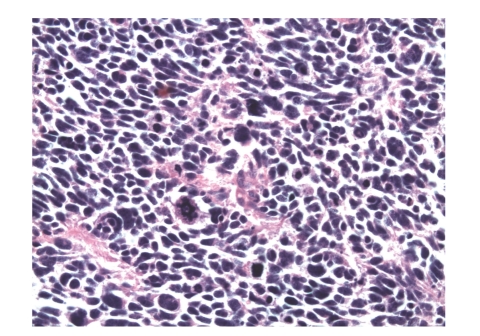
In the higher magnification, nuclear moldings with many abnormal mitosis, apoptotic cells, and necrosis are seen. (HE) ×400.

The patient was taken to the operating room for a laparoscopic exploration and surgical resection. Exploratory laparoscopy yielded no evidence of metastatic disease. The gallbladder was excised en bloc with a subsegmental liver resection of segment 4B. Pathology was consistent with a poorly differentiated neuroendocrine carcinoma with extensive lymphovascular invasion (Figures 2 and 3). The Azzopardi phenomenon, a feature of small cell carcinoma, was present (Figure 4). An immunohistochemical analysis confirmed the diagnosis (Table 1).

The patient recovered from the intervention without complication. He has since received a six-week course of 54 Gy of radiotherapy with a concurrent chemotherapy regimen of cisplatin and etoposide. Currently, this individual is eight months out from his initial operation and is doing well.

## 3. Case 2

A 41-year-old female presented to an outside institution with symptoms consistent with biliary dyskinesia for which an uncomplicated laparoscopic cholecystectomy was performed. The pathology returned with an incidental finding of a well-differentiated neuroendocrine carcinoma of the cystic duct. The mass was 0.4 centimeters in largest diameter with the proximal margin of the cystic duct positive for malignancy. An MRI and abdominal CT scan performed at that time were negative for any evidence of residual tumor or metastatic disease. She was subsequently referred to this institution for additional surgical treatment.

Following the completion of a negative metastatic work up with laboratory analyses and an Octreoscan, the patient was taken to the operative suite for re-exploration. The cystic duct remnant was resected at its origin, and a wide portal lymphadenectomy was completed. When the frozen pathology returned with no evidence of residual tumor, the operation was concluded.

The patient tolerated the procedure well. Pathology findings were negative for any additional tumor. She has since been treated with adjuvant chemotherapy by medical oncology. A repeat octreotide scan two months postoperatively documented no recurrent or metastatic disease.

## 4. Discussion

Gallbladder carcinoma is the fifth most common gastrointestinal malignancy with 7000 new cases diagnosed in the United States each year [1]. The preponderance of these tumors is adenocarcinoma [4, 5]. It is a disease with few well-described risk factors. These include choledochal cysts, gallstones larger than three centimeters, and chronic states of inflammation [2]. Due to the lack of systemic symptoms, it often presents at an advanced stage with only ten percent of cases confined to the gallbladder wall at the time of diagnosis [8]. It is because of this insidious presentation that the overall survival rate is less than 5%. To date, there has been minimal research conducted as to the medical treatment of this disease with most trials being underpowered or overinclusive with multiple tumor types enrolled in the trial. Adjuvant chemotherapy is often used but is without a well-defined protocol. Therapeutic interventions are currently limited to complete surgical resection with negative margins. Unfortunately, resection is feasible in no more than 25% of patients due to the advanced nature of their disease at presentation.

Neuroendocrine carcinomas represent an even smaller percentage of the general surgeon's practice. Carcinoid tumors comprise the majority of this pathology, with as many as 68% originating from the gastrointestinal tract [9]. The varied presentation of this tumor is based on its embryologic origin and may include mass effect, bleeding, or the classic carcinoid syndrome. The gallbladder small cell neuroendocrine tumor is an exceedingly rare malignancy comprising 0.2% of all gastrointestinal carcinoids [10]. It is typically a nonfunctional tumor without overt clinical symptoms secondary to secretion of biologically active peptides [9]. Furthermore, it is often discovered late in the disease process when adjacent organ systems have been violated or the biliary system becomes obstructed. Consequently, the prognosis is poor with survival rates worse than other gallbladder malignancies. 

There have been limited discussions of this pathology in the literature to date, principally in the form of case reports or small case series. It is a disease that most commonly affects an elderly, female population [11]. Similar to adenocarcinoma, there is a strong association between cholelithiasis and this tumor. Interestingly, there are no neuroectodermal cells in the gallbladder mucosa [12]. This had lead investigators to postulate that the small cell carcinoma arises from metaplastic epithelium of the gallbladder wall [13]. This hypothesis is supported by the presence of metaplasia in the gallbladder with chronic cholecystitis [14]. Distant disease at presentation is present as much as 75% of the time and is most often located in the liver and adjacent lymph nodes [5]. 

Due to the scarcity of this neoplasm, diagnostic criteria in the form of radiologic findings and preoperative tumor markers are poorly described. Furthermore, given the insidious nature of the mass, diagnosis can be difficult without a histologic analysis. For instance, in the first patient, the MRI was notable for a large, infiltrating mass. There were no notable abnormalities in the laboratory work up including CEA and CA19-9. In the second subject, it was not until after the therapeutic cholecystectomy for acute cholecystitis that the mass was incidentally discovered. Even with imaging, it is often not until the histopathologic analysis is complete that the diagnosis of a neuroendocrine tumor is reached.

Therapeutic options for this pathology are often limited due to the advanced nature of the disease at diagnosis. Surgical excision remains the best option for a cure. However, the necessity of a radical resection and the extent of surrounding tissue removed are debated in the literature [15]. In those patients not amenable to a curative operation-different chemotherapy regimens are described [4, 6, 16, 17]. Patients treated with surgical excision and adjuvant chemotherapy have an increased median survival from 4.5 to 13 months [11]. With that said, no randomized, blinded trials have been performed to validate one regimen as the gold standard treatment.

## 5. Conclusion

Gallbladder neuroendocrine carcinomas remain an exceptionally rare malignancy with a strikingly poor prognosis. Early diagnosis with prompt surgical intervention provides the patient with the best long-term outcome. Adjuvant chemotherapy provides a definable survival advantage to the patient but is without a well-defined standard of care protocol.

## Figures and Tables

**Figure 5:**
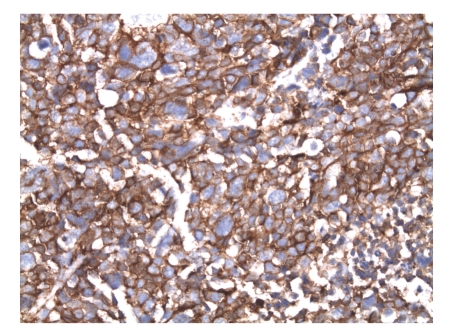
The Azzopardi phenomenon: basophilic dark blue DNA/nuclear chromatin liberated from dead cells coats vessel walls. (HE) ×400.

**Figure 6:**
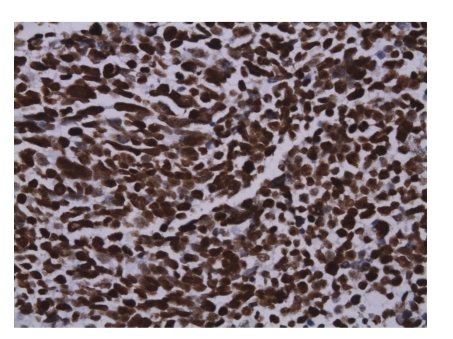
The tumor cells are strongly diffuse positive for chromogranin A (Immunohistochemical stain, original magnification, ×400). (IHC).

**Table 1:** 

Immunohistochemical stains	Tumor response to stain
Synaptophysin	Positive
Chromogranin	Positive
CD56	Positive
CD57	Positive
Cytokeratin AE1/AE3	Negative
TTF1	Negative
CD45	Negative
Ki-67	Positive (>90% Tumor cells)
